# Extracellular HMGB1 Induced Glomerular Endothelial Cell Injury via TLR4/MyD88 Signaling Pathway in Lupus Nephritis

**DOI:** 10.1155/2021/9993971

**Published:** 2021-12-21

**Authors:** Tian Yu, Feng Xiaojuan, Liu Jinxi, Miao Xinyan, Xu Jie, Tian Yuexin, Liu Qingjuan, Zhang Wei, Gu Cunyang, Huang Jie, Wu Lunbi, Zhao Hang, Liu Shuxia, Guo Huifang

**Affiliations:** ^1^Department of Pathology, Key Laboratory of Kidney Diseases of Hebei Province, Center of Metabolic Diseases and Cancer Research, Hebei Medical University, Zhongshan East Road No. 361, Shijiazhuang, China 050017; ^2^Department of Rheumatology, The Second Hospital of Hebei Medical University, Heping West Road No. 252, Shijiazhuang, China 050000

## Abstract

Previously, our study showed that HMGB1 was significantly elevated in the blood and located in the glomerular endothelium in LN patients. But whether extracellular HMGB1 is involved in the injury of glomerular endothelial cells (GECs) in LN still needs further investigation. Firstly, we detected the levels of SDC-1, VCAM-1, and proteinuria in LN patients and MRL/lpr mice and analyzed their correlations. Then, HMGB1 and TLR4/MyD88 were inhibited to observe the shedding of glycocalyx and injury of GECs in vivo and in vitro. Our results showed that HRGEC injury and SDC-1 shedding played an important role in the increase of permeability and proteinuria formation in LN. Additionally, inhibition of extracellular HMGB1 and/or downstream TLR4/MyD88/NF-*κ*B/p65 signaling pathway also alleviated GEC monolayer permeability, reduced the shedding of the glomerular endothelial glycocalyx, improved the intercellular tight junction and cytoskeletal arrangement, and downregulated the NO level and VCAM-1 expression. These results suggested that extracellular HMGB1 might involve in GEC injury by activating the TLR4/MyD88 signaling pathway in LN, which provided novel insights and potential therapeutic target for the treatment of lupus nephritis.

## 1. Introduction

Systemic lupus erythematosus (SLE) is a chronic autoimmune disease that often involves multiple tissues and organs. Lupus nephritis (LN) is one of the most serious complications of SLE and a major risk factor for morbidity and mortality [1]. Though overall survival of LN patients has steadily improved in these years [2, 3], still 4.3-10.1% rate of patients develops to end-stage renal disease (ESRD) [4].

Proteinuria is one of the most representative manifestations of LN, and the key pathological feature is the damage of the glomerular filtration barrier (GFB). Glomerular endothelium is the first layer of GFB, including glomerular endothelial cells (GECs), the glycocalyx covering the surface of the GECs, transcellular fenestrations, and tight junctions among cells [5]. In brief, glomerular endothelial dysfunction contributes to hypertension, edema, and proteinuria [6]. Of note, cooccurrence of podocyte and GEC injury could lead to nephrotic syndrome in proliferative LN [7]. Importantly, the endothelial glycocalyx plays a crucial role in vascular permeability, adhesion of leukocytes and platelets, the regulation of shear stress, and inflammatory processes [8]. However, the precise mechanism of GEC dysfunction in the pathogenesis of LN needs further research.

High mobility group box 1 (HMGB1) is a nonhistone nuclear protein that is ubiquitously expressed in the nucleus. Once passively released or actively secreted, extracellular HMGB1 would mediate inflammation, immunity, cell migration, invasion, and so on. At present, the level of extracellular HMGB1 has been proved elevated in SLE patients and correlated with disease activity [9, 10]. Our previous research has confirmed that HMGB1 was one of the most crucial cytokines in LN and contributed to mesangial cell proliferation and extracellular matrix deposition [11, 12]. In addition, we also found that extracellular HMGB1 was not only predominantly located in the glomerular mesangial cell but also differentially expressed in endothelial cells of glomeruli in LN. Other research has revealed that HMGB1 was contributed to vascular endothelial inflammation in hyperhomocysteinemia [13]. Usually, extracellular HMGB1 induced the production of inflammatory cytokines or mediated cell injury by binding to cell surface receptors such as Toll-like receptors (TLRs) and triggering related intracellular pathways [14, 15]. However, whether HMGB1 is involved in vascular endothelial injury of LN and the possible mechanism are poorly understood. In the present study, we confirmed again the effect of endothelial injury on proteinuria formation and investigated whether extracellular HMGB1 induced endothelial cell injury by activating downstream signaling pathway TLR4/MyD88. Our results showed that inhibiting the HMGB1 and TLR4/MyD88 signaling pathway alleviated glycocalyx shedding and GEC injury in MRL/lpr mice and LN plasma-stimulated HRGECs, which will provide a new direction for therapeutic target of endothelial injury and proteinuria in LN.

## 2. Materials and Methods

### 2.1. Patients and Samples

This study was approved by the Second Hospital of Hebei Medical University. All participants provided informed consent and permission.

Thirty patients aged 18-55 years with renal biopsy-confirmed LN were recruited from the Department of Nephrology at the Second Hospital of Hebei Medical University between 2015 and 2018. Serum, urine, and renal biopsy samples were collected. In addition, 19 age- and sex-matched healthy volunteers were used as controls. Additionally, control renal tissues (pathologically confirmed as normal kidney tissues) were obtained from eight patients undergoing nephrectomy due to lipomyoma, leiomyoma, or kidney cancer. None of the patients had other autoimmune diseases, diabetic nephropathy, hypertensive nephropathy, or infection. The serum and urine samples were frozen at -80°C until analysis, while the kidney tissues were fixed with 4% formaldehyde and embedded in paraffin for immunofluorescence (IF) and immunohistochemistry (IHC) staining.

Furthermore, five plasma samples were collected from patients who were diagnosed with LN in the Inpatient Department of Nephrology at the Second Hospital of Hebei Medical University from 2018 to 2019, without the initiation of immunosuppressive therapy, infections, or other complications, but underwent therapeutic plasma exchange. Furthermore, five plasma samples from age- and sex-matched healthy volunteers were acquired as controls.

### 2.2. Reagents and Antibodies

Monoclonal rabbit anti-SDC-1, anti-VCAM-1, anti-MyD88, anti-TLR4, anti-p-I*κ*B*α*-Ser36 antibodies and monoclonal mouse anti-CD31 antibodies were purchased from Abcam (Cambridge, UK). Monoclonal rabbit anti-vascular endothelial- (VE-) cadherin and anti-NF-*κ*B/p65 antibodies were bought from Cell Signaling Technology (Boston, MA, USA). Tetramethylrhodamine- (TRITC-) phalloidin (which binds to F-actin) was from Solarbio (Beijing, China), and 4′,6-diamidino-2-phenylindole (DAPI) was from SouthernBiotech (Birmingham, USA). Anti-HMGB1 antibody was from Wanleibio (Shenyang, China), and control IgG antibody was from Beyotime (Shanghai, China). Glycyrrhizin (GLY; an HMGB1 inhibitor) was obtained from JK Scientific (Beijing, China). TAK242 (a TLR4 inhibitor), ST2825 (a MyD88 inhibitor), and SN50 (an inhibitor of NF-*κ*B/p65 translocation) were purchased from MedChemExpress (NJ, USA). Fluorescein isothiocyanate-labeled bovine serum albumin (FITC-BSA) was purchased from Solarbio (Beijing, China). A human SDC-1 enzyme-linked immunosorbent assay (ELISA) kit was purchased from Abcam (Cambridge, UK), and a mouse SDC-1 ELISA kit and VCAM-1 ELISA kit were purchased from Zcibio (Shanghai, China). A Total Nitric Oxide (NO) Assay Kit was purchased from Beyotime Biotechnology (Shanghai, China).

### 2.3. Cell Culture and Groups

Primary human renal GECs (HRGECs), which were isolated from human kidneys and identified by IF staining with anti-von Willebrand factor/factor VII and CD31 antibodies, were purchased from ScienCell Research Laboratories, Inc. (San Diego, CA, USA). Cell dishes were coated with fibronectin (2 *μ*g/cm^2^) in a 37°C incubator overnight. Thereafter, the HRGECs were cultured in the cell dishes with endothelial cell medium (San Diego, CA, USA) supplemented with 5% fetal bovine serum (FBS), 1% endothelial cell growth supplement, and 1% penicillin/streptomycin solution. Before use, the cells were synchronized in a serum-free medium for 12 h.

The group is as follows:

1. To evaluate the effect of LN plasma on the permeability of HRGEC monolayers and the TLR4/MyD88/NF-*κ*B signaling pathway, cells were randomly divided into the control or LN groups and, respectively exposed to control plasma or LN plasma for 0, 1, 2, 4, 6, 12, 18, and 24 h. The permeability of HRGEC monolayers was assessed based on transendothelial electrical resistance (TEER) values and the passage of FITC-BSA across monolayers in transwell inserts. Western blotting and IF were used to detect the expression of TLR4, MyD88, p-I*κ*B*α*-Ser36, NF-*κ*B/p65, and VCAM-1 protein
2. To investigate the role of HMGB1, TLR4, MyD88, and NF-*κ*B/p65 in the LN plasma-induced permeability of HRGEC monolayers, cells were randomly divided into eight groups named as control, LN, LN+GLY, LN+GLY+TAK, LN+TAK, LN+ST2825, LN+SN50, and LN+DMSO groups. The first two groups were exposed to control plasma or LN plasma, respectively. The cells in the LN+GLY, LN+GLY+TAK, LN+TAK, LN+ST2825, and LN+SN50 groups were, respectively, pretreated with GLY (150 *μ*g/mL), GLY plus TAK242 (1 *μ*mol/L), TAK242, ST2825 (2 *μ*mol/L), SN50 (2 *μ*mol/L), or DMSO for 30 min, then exposed to LN plasma for 24 h. TEER values and permeability of FITC-BSA were monitored in transwell inserts. The cell supernatants and lysates were collected for ELISA, western blotting, and NO detection. In addition, the cells were analyzed with IF staining to assess the expression of VE-cadherin, F-actin, SDC-1, and NF-*κ*B/p65
3. To further explore the effect of extracellular HMGB1, anti-HMGB1 antibody was used. Firstly, the cells were pretreated with anti-HMGB1 antibody or control IgG for 30 min. Then, the cells were exposed to control plasma or LN plasma, respectively. At 24 h, the cells were collected and TEER values, permeability of FITC-BSA, ELISA, western blotting, NO detection, and IF were carried out as above

### 2.4. Animal Experiments

MRL/lpr and MRL/MPJ mice (with equal numbers of males and females) were purchased from Jackson Laboratory (Bar Harbor, USA). The animal protocols were approved by the Institutional Animal Care and Use Committee of Hebei Medical University (approval ID: HebMU 20080026).

Six 16-week-old MRL/MPJ mice were used as the control group. Thirty aged-matched MRL/lpr mice (a mouse model used to study SLE) were randomly divided into the following five groups (*n* = 6 per group): LN, LN+GLY, LN+NS, LN+TAK242, and LN+DMSO. The mice in LN+GLY and LN+NS groups were intraperitoneally injected with GLY (10 mg/kg, every day) or normal saline (NS, every day), while mice in LN+TAK242 and LN+DMSO groups were intraperitoneally injected with TAK242 (10 mg/kg, twice a week) or DMSO (approximately equal volume to the TAK242 solution, twice a week), respectively. At 24 weeks old, the mice were sacrificed and urine, plasma, and renal tissues were collected for further analysis.

Meanwhile, the renal cortex was prepared to observe the endothelial glycocalyx by transmission electron microscopy (TEM), which will be described in Section 2.7 in detail.

### 2.5. ELISA

The levels of SDC-1 and VCAM-1 in human serum, mouse plasma, and HRGEC supernatant were measured by ELISA kits in accordance with the manufacturers' instructions. The absorbance was measured with a spectrophotometer at 450 nm.

### 2.6. IF and IHC Staining

IF and IHC staining were carried out according to our previous study [11]. The concentration of primary antibodies against SDC-1, VCAM-1, CD31, TLR4, MyD88, NF-*κ*B/p65, VE-cadherin, and NF-*κ*B/p65 was 1 : 200. The expression of proteins was quantified by digital image analysis using Image-Pro Plus 5.0 software (Media Cybernetics, Silver Spring, MD, USA) based on the integrated optical density (IOD) of the positively stained region.

### 2.7. TEM

Mice were anesthetized, and an incision was made in the right atrial appendage and perfused with a solution composed of 2% lanthanum nitrate and 0.2 mol/L sodium dimethylarsenate. When the kidneys became pale, the mice were perfused with a fixative (containing 2% lanthanum nitrate, 2% glutaraldehyde, 2% paraformaldehyde, and 0.2 mol/L sodium cacodylate). Then, approximately 1 mm^3^ renal cortex of different groups was immersed again. Washed in 0.1 mol/L phosphate buffer, postfixed in 1% osmium tetroxide, and embedded in pure acetone solution, the ultrathin sections were stained with uranyl acetate and lead citrate, then examined by TEM (HT-7700, Hitachi, Japan).

### 2.8. Determination of NO Level

Mouse renal cortex and HRGECs in different groups were lysed, and the level of NO was measured using the Total NO Assay Kit according to the manufacturer's instructions.

### 2.9. Transendothelial Electrical Resistance (TEER)

According to the manufacturer's recommendation, transwell chambers (Corning, New York, USA) were incubated with bovine fibronectin at 37°C overnight. Thereafter, 5 × 10^5^ HRGECs were added to the upper chamber (0.4 *μ*m pore size) and 1.5 mL total endothelial cell medium was added to the lower chamber. When HRGECs reached confluence and formed an integrated monolayer, the TEER of transwell inserts was measured and the mean value was expressed in *Ω*·cm^2^ after the value of a blank (cell-free) insert was subtracted. Data were normalized to the relevant controls.

### 2.10. HRGEC Monolayer Permeability Assay

HRGEC monolayer permeability was determined using FITC-BSA. After HRGECs in the upper transwell chamber reached confluence, 0.1 mL FITC-BSA (5 mg/mL) and 0.3 mL equimolar unlabeled BSA were added to the upper and lower chamber, respectively. According to experimental design, the medium was collected and the fluorescence was measured using a microplate fluorescence reader (Berthold, Germany) with filter settings of 495 nm (excitation) and 520 nm (emission).

The permeability coefficient (Pa) associated with BSA was then used to indicate the permeability of the HRGEC monolayer. The formula to calculate Pa was as follows: Pa = [*A*] (1/*A*) (*v*/[*L*]), where [*A*] is the fluorescence intensity (FITC-BSA) in the lower chamber, *A* is the bottom area of the upper chamber, *v* is the volume of solution in the lower chamber, and [*L*] is the fluorescence intensity in the upper chamber. Permeability (*P*Δ), % = (experimental group Pa/control group Pa) × 100%. Experiments were repeated five times.

### 2.11. Western Blotting

Protein extraction and western blotting were performed as previously described [16]. Briefly, HRGECs were collected and protein was extracted. Then, the protein was separated by 10% sodium dodecyl sulfate-polyacrylamide gel electrophoresis (SDS-PAGE) and transferred to polyvinylidene difluoride (PVDF) membranes (Millipore, USA). Hereafter, the membranes were blocked with 5% BSA followed by incubation with rabbit anti-*β*-actin (1 : 1000), anti-TLR4 (1 : 1000), anti-MyD88 (1 : 1000), anti-NF-*κ*B/p65 (1 : 1000), or anti-p-I*κ*B*α*-Ser36 (1 : 2000) antibody overnight. Thereafter, horseradish peroxidase- (HRP-) conjugated goat anti-rabbit IgG secondary antibody (Proteintech, Wuhan, China) (1 : 5000) was added for 2 h at room temperature. After washing with Tris-buffered saline with Tween 20 (TBST), the signals were detected using a LI-COR Odyssey Infrared Imaging System (Lincoln, NE, USA). All experiments were repeated at least three times.

### 2.12. Statistical Analysis

SPSS 21.0 (SPSS, Inc., Chicago, IL, USA) was used for data analysis. The quantitative data are expressed as the mean ± standard deviation (SD). The statistical significance was performed by one-way analysis of variance (ANOVA) and the Student–Newman–Keuls test. The Spearman rank correlation test was used to assess the relationships between two-group parameters. A *p* value < 0.05 was considered statistically significant.

## 3. Results

### 3.1. Glycocalyx Shedding of Glomerular Endothelial Cell Was Positively Associated with Proteinuria in LN

**Figure 1:**
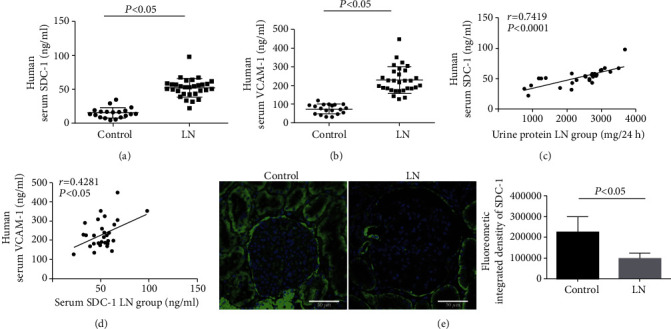
The level of SDC-1 was associated with the levels of VCAM-1 and proteinuria in LN patients. (a, b) ELISA showing the levels of serum SDC-1 and VCAM-1 in the control and LN patients. (c, d) The level of serum SDC-1 in LN patients was correlated with 24 h proteinuria (*r* = 0.7419, *p* < 0.0001) and serum VCAM-1 (*r* = 0.4281, *p* < 0.05). (e) The expression of SDC-1 protein in renal glomeruli cells of LN patients was detected by IF staining. SDC-1: syndecan-1; VCAM-1: vascular cell adhesion molecule-1; ELISA: enzyme-linked immunosorbent assay; IF: immunofluorescence; LN: lupus nephritis.

Firstly, the serum levels of SDC-1 (a core protein of the glycocalyx) and VCAM-1 (an adhesion molecule and a marker of endothelial cell activation) in LN patients and controls were detected by ELISA. As shown in Figures 1(a) and 1(b), the levels of SDC-1 and VCAM-1 were remarkably increased in LN patients compared to the control group. More importantly, the serum SDC-1 level was significantly positively correlated with the levels of proteinuria and serum VCAM-1 (*r* = 0.7419, 0.4281, Figures 1(c) and 1(d)). In addition, IF staining showed that the positive signal of SDC-1 protein was mainly located in the membranes of glomeruli intrinsic cells and significantly decreased in glomeruli of LN patients (Figure 1(e)).

**Figure 2:**
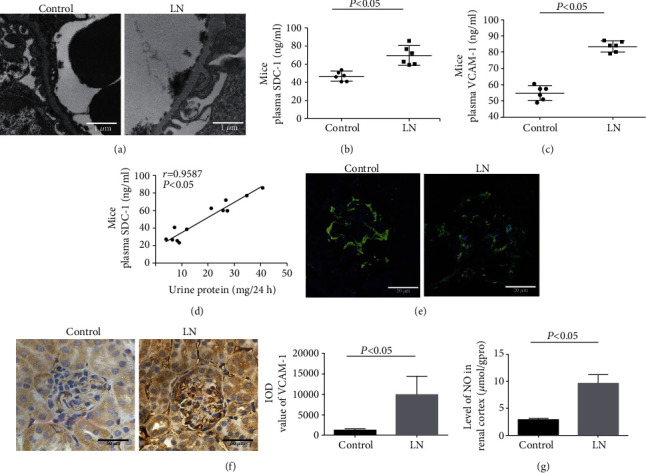
Glomerular endothelial glycocalyx shedding was associated with proteinuria in MRL/lpr mice. (a) Glomerular endothelial glycocalyx of mice was observed by TEM. (b, c) The level of SDC-1 and VCAM-1 in plasma of control and LN mice was measured by ELISA (*n* = 6). (d) Correlation analysis of plasma SDC-1 level and 24 h proteinuria in mice (*r* = 0.9587, *p* < 0.05). (e) IF of SDC-1 (red) and CD31 (green) in glomeruli of control and LN mice. (f) IHC of VCAM-1 in mouse glomeruli of control and LN groups. (g) The level of NO in the renal cortex of control and LN mice was detected (*n* = 6). NO: nitric oxide.

Furthermore, TEM revealed that the glycocalyx with of glomerular endothelial cells was significantly thinner in the MRL/lpr mice compared to control mice (Figure 2(a)). Consistently, the plasma levels of SDC-1 and VCAM-1 were obviously upregulated in MRL/lpr mice compared to control mice (Figures 2(b) and 2(c)). Remarkably, the level of SDC-1 was positively correlated with the level of proteinuria in MRL/lpr mice (*r* = 0.9587, Figure 2(d)). To further observe the expression of SDC-1 in GECs, IF staining of CD31 (an endothelial cell marker, green) and SDC-1 (red) was conducted. As illustrated in Figure 2(e), SDC-1 was significantly downregulated in the GECs (CD31-positive cells) of MRL/lpr mice compared to control mice. However, compared to control mice, IHC showed that VCAM-1 was significantly upregulated in the glomeruli of MRL/lpr mice (Figure 2(f)). Additionally, the level of NO in the renal cortex was notably higher in MRL/lpr mice than that in control mice (Figure 2(g)).

In summary, glycocalyx shedding was contributed to proteinuria in the pathogenesis of LN.

### 3.2. GEC Injury and SDC-1 Shedding Played an Important Role in the Increase of Permeability in LN

**Figure 3:**
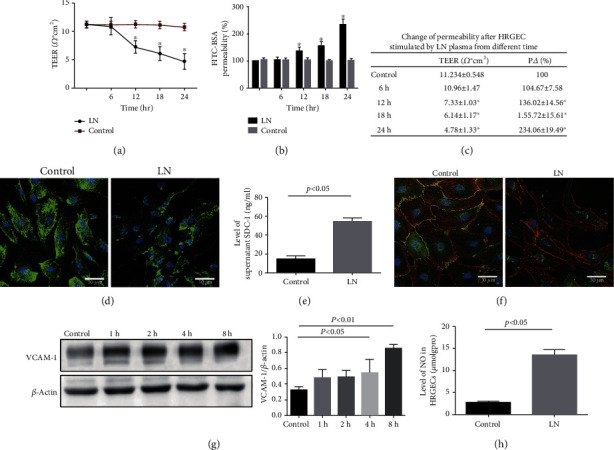
HRGEC injury contributed to the upregulation of glomerular filtration membrane permeability in LN. (a) TEER of HRGECs stimulated by LN plasma at different time was measured (*n* = 5). (b) HRGEC glomerular filtration membrane permeability was assessed by the FITC-BSA method (*n* = 5). (c) Change in TEER and glomerular filtration membrane permeability of HRGEC. (d) IF staining showing the expression of SDC-1 (green) in HRGECs. (e) The level of SDC-1 in supernatant was measured by ELISA (*n* = 5). (f) Colocalization of VE-cadherin (green) and F-actin (red) in HRGECs was determined by IF staining. (g) Western blotting of VCAM-1 in LN plasma-stimulated HRGECs. (h) The level of NO in HRGECs was measured (*n* = 5). *p* < 0.05 vs. control group. TEER: transendothelial electrical resistance.

To further explore the possible mechanism of GEC dysfunction in LN, HRGECs were exposed to LN plasma. As shown in Figures 3(a)–3(c), the TEER value at 24 h in the LN group was 4.78 ± 1.33 *Ω*·cm^2^, which was significantly lower than that in the control group (11.23 ± 0.58 *Ω*·cm^2^). Additionally, the permeation of FITC-BSA at 24 h was 234.06 ± 19.49% in the LN group, which was significantly higher than that in the control group (*p* < 0.05), suggesting that LN plasma increased the HRGEC monolayer permeability.

Next, to confirm the underlying mechanism of increased glomerular filtration membrane permeability in LN, IF staining and ELISA were used. As shown in Figure 3(d), SDC-1 positive staining was located in the cytomembrane and cytoplasm of HRGECs, and the expression was distinctly downregulated in the LN group. Moreover, the SDC-1 level in the supernatant was remarkably increased in the LN group compared to the control group (Figure 3(e)).

In addition, IF staining of VE-cadherin (green) and F-actin (red) showed that the distribution of VE-cadherin was continuous and F-actin was regularly arranged in the control group, while the HRGECs shrank and the distribution of VE-cadherin was discontinuous and the F-actin arrangement was disordered, showing a sawtooth-like structure (Figure 3(f)) in the LN group, which indicated that LN plasma induced structural injury of GECs.

Furthermore, the result of western blotting showed that the expression of VCAM-1 protein in the LN group was significantly increased at 8 h compared to the control group (Figure 3(g)). Importantly, the level of NO was also significantly increased in the LN group (Figure 3(h)).

Taking together, LN plasma indeed induced GEC injury and SDC-1 shedding, which played an important role in the increased permeability of GEC in the pathogenesis of LN.

### 3.3. Extracellular HMGB1 Mediated GEC Injury and SDC-1 Shedding in LN

**Figure 4:**
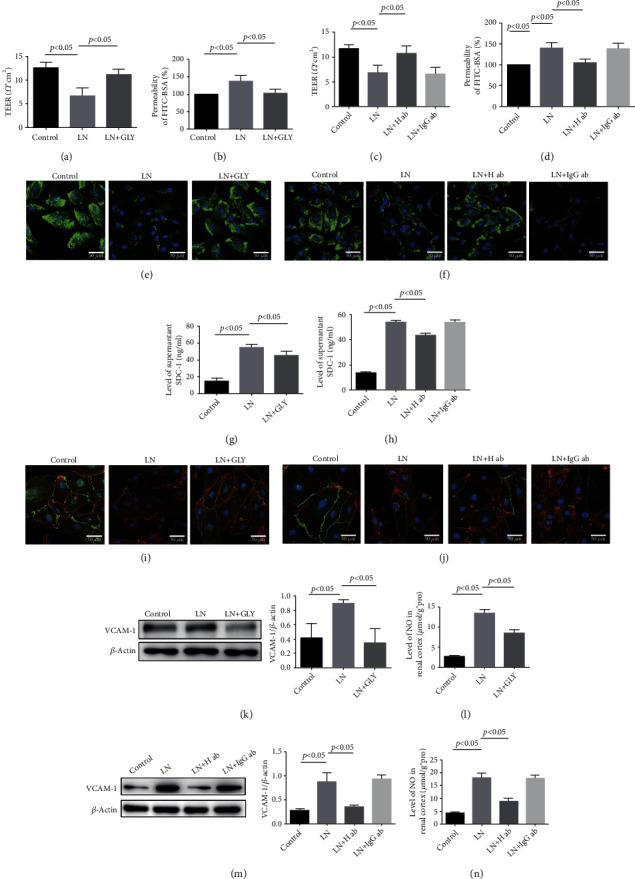
GLY and anti-HMGB1 antibody alleviated the GEC injury in LN. (a–d) TEER and permeability of HRGEC in control, LN, LN+GLY, LN+H ab, and LN+IgG ab groups (*n* = 5). (e, f) SDC-1 (green) in HRGECs was detected by IF staining. (g, h) The level of SDC-1 in supernatant was measured by ELISA (*n* = 5). (i, j) Colocalization of VE-cadherin (green) and F-actin (red) in HRGECs was detected by IF staining. (k, m) The expression of VCAM-1 in HRGECs was measured by western blotting. (l, n) The level of NO in HRGECs (*n* = 5). GLY: glycyrrhizin; H ab: anti-HMGB1 antibody; IgG ab: control IgG.

Our previous research has shown that HMGB1 was an important cytokine in the pathogenesis of LN and contributed to mesangial cell proliferation and proteinuria formation [11]. To confirm whether extracellular HMGB1 contributed to GEC injury in LN, GLY (a HMGB1 inhibitor) and anti-HMGB1 antibody were used. As illustrated in Figures 4(a)–4(d), GLY and anti-HMGB1 antibody separately relieved the LN plasma-induced HRGEC monolayer hyperpermeability at 24 h. IF staining and ELISA also showed that inhibition of HMGB1 reversed the low expression of SDC-1 in HRGECs and reduced the supernatant level of SDC-1 (Figures 4(e)–4(h)) induced by LN plasma. Furthermore, the expression of VE-cadherin and the rearrangement of F-actin were significantly improved in LN+GLY and LN+anti-HMGB1 groups compared to the LN group (Figures 4(i) and 4(j)). Similarly, VCAM-1 protein expression and NO level were significantly downregulated (Figures 4(k)–4(n)).

**Figure 5:**
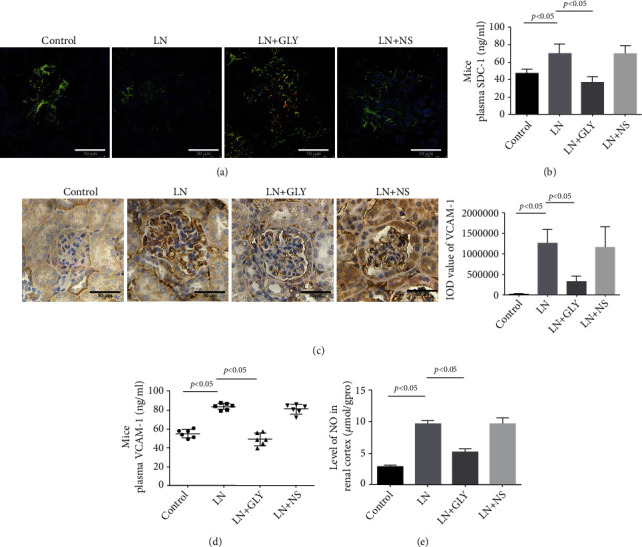
GLY treatment improved the injury of GECs in MRL/lpr mice. (a) Colocalization of CD31 (green) and SDC-1 (red) in mouse glomeruli cells was detected by IF staining. (b) The level of mouse plasma SDC-1 measured by ELISA (*n* = 6). (c) IHC showed the expression of VCAM-1 in mouse glomeruli. (d) The level of VCAM-1 in mouse plasma assessed by ELISA (*n* = 6). *p* < 0.05 vs. LN group. (e) The level of NO in mice renal cortex (*n* = 6).

*In vivo*, the shedding of SDC-1 in GECs and the level of SDC-1 in the plasma were relieved in MRL/lpr mice treated with GLY (Figures 5(a) and 5(b)). Additionally, IHC staining and ELISA revealed that GLY treatment downregulated the VCAM-1 expression not only in glomeruli but also in plasma (Figures 5(c) and 5(d)), as well as reversed the enhanced level of NO in the renal cortex of MRL/lpr mice (Figure 5(e)).

### 3.4. Extracellular HMGB1 Mediated GEC Injury by Activating the TLR4/MyD88/NF-*κ*B/p65 Pathway in LN

**Figure 6:**
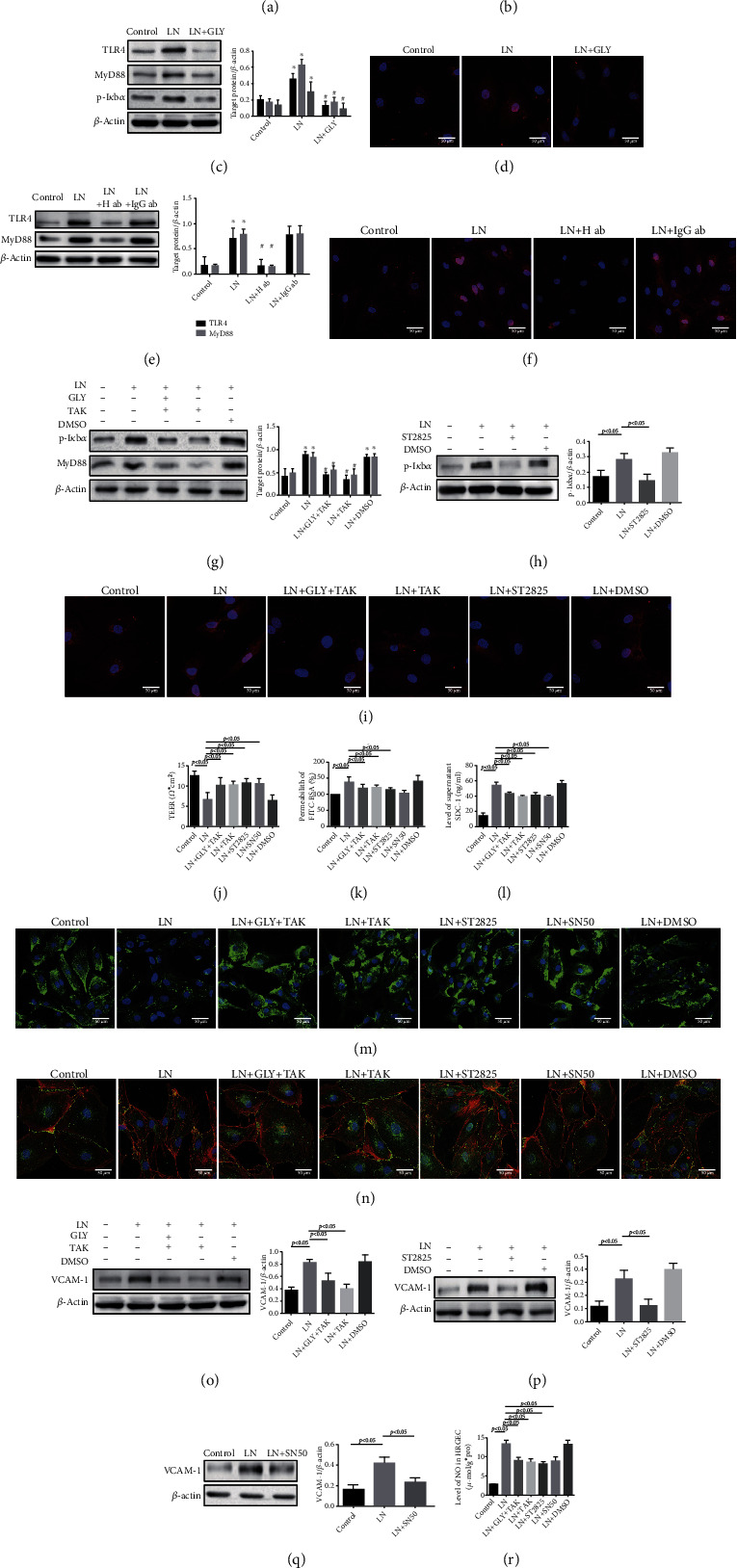
Inhibition of the TLR4/MyD88/NF-*κ*B/p65 pathway relieved GEC injury in HMGB1-induced LN. (a) Western blotting showed the expression of TLR4, NF-*κ*B/p65, p-I*κ*B*α*, and MyD88 in HRGECs cultured with LN plasma. (b) IF staining for NF-*κ*B/p65 (red) in HRGECs cultured with LN plasma. (c, e, g, and h) Western blotting of TLR4, MyD88, and p-I*κ*B*α* in LN plasma-stimulated HRGECs pretreated with different inhibitors. (d, f, and i) IF staining of NF-*κ*B/p65 (red) in LN plasma-stimulated HRGECs pretreated with different inhibitors. (j, k) TEER and permeation in LN plasma-stimulated HRGECs pretreated with different inhibitors (*n* = 6, *p* < 0.05). (l) The level of SDC-1 in supernatant of LN plasma-stimulated HRGECs pretreated with different inhibitors measured by ELISA. (m) IF staining of SDC-1 in LN plasma-stimulated HRGECs pretreated with different inhibitors. (n) IF staining of VE-cadherin (green) and F-actin (red) in LN plasma-stimulated HRGECs pretreated with different inhibitors. (o–q) Western blotting of VCAM-1 in LN plasma-stimulated HRGECs pretreated with different inhibitors. (r) The level of NO in LN plasma-stimulated HRGECs pretreated with different inhibitors. ^∗^*p* < 0.05 vs. control group; ^#^*p* < 0.05 vs. LN group.

To explore the precise mechanism of extracellular HMGB1-induced injury of GEC, TLR4 (an important receptor of HMGB1) and related signal were detected. As shown in Figure 6(a), the expression of TLR4, MyD88, p-I*κ*B*α*, and NF-*κ*B/p65 protein was upregulated in HRGECs exposed to LN plasma at 2, 4, and 8 h, respectively (vs. control group). Histologically, NF-*κ*B/p65 was mainly located in the cytoplasm in the control group, whereas nuclear translocation was significantly increased at 8 h in the LN group (Figure 6(b)). Pretreatment of HRGECs with GLY, anti-HMGB1 antibody, TAK242 (a TLR4 inhibitor), or ST2825 (a MyD88 inhibitor), respectively, downregulated TLR4, MyD88, and p-I*κ*B*α* protein expression and reduced NF-*κ*B/p65 nuclear translocation induced by LN plasma (Figures 6(c)–6(i)). Furthermore, inhibition of TLR4, MyD88, and NF-*κ*B/p65 translocation (using SN50) relieved the LN plasma-induced HRGEC monolayer hyperpermeability (Figures 6(j) and 6(k)), prevented SDC-1 shedding from HRGECs (Figures 6(l) and 6(m)), improved the expression of VE-cadherin and F-actin (Figure 6(n)), and reversed the increased levels of VCAM-1 and NO (Figures 6(o)–6(r)). However, there were no significant differences irrespectively of whether the HRGEC cells were pretreated with GLY and TAK242 together or alone.

**Figure 7:**
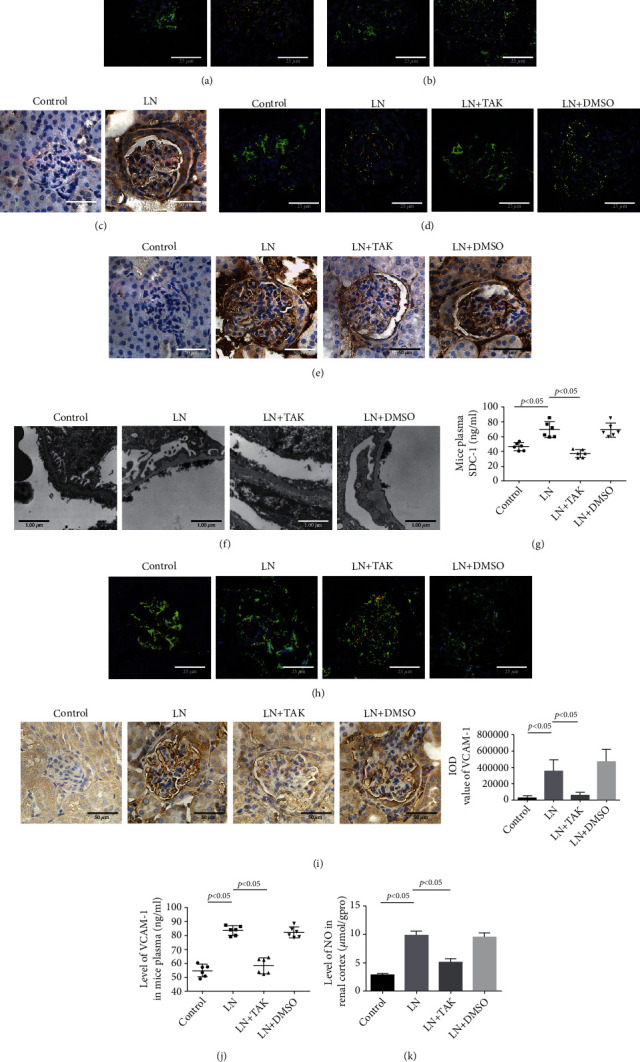
TAK242 alleviated the GEC injury in MRL/lpr mice. (a) IF staining of TLR4 (red) and CD31 (green) in the renal glomeruli cells of control and LN groups. (b) IF staining of MyD88 (red) and CD31 (green) in the renal glomeruli cells of control and LN groups. (c) IHC of NF-*κ*B/p65 in the renal glomeruli cells of control and LN groups. (d) IF staining of MyD88 (red) and CD31 (green) in the renal glomeruli cells of mice. (e) IHC of NF-*κ*B/p65 in the renal glomeruli of mice. (f) TEM showed the glomerular endothelial glycocalyx of mice. (g) The level of SDC-1 in mouse plasma detected by ELISA. (h) IF staining of SDC-1 (red) and CD31 (green) in the renal glomeruli cells of mice. (i) IHC of VCAM-1 in the mouse renal glomeruli cells of mice. (j) The level of VCAM-1 in the mouse plasma assessed by ELISA. (k) The level of NO in the renal cortex of mice. ^∗^*p* < 0.05 vs. control group. GEC: glomerular endothelial cell; TAK: TAK242.

In the MRL/lpr mice, TLR4 (red) and MyD88 (red) expression significantly increased in the cytoplasm of GECs (green) (CD31-positive cells) and NF-*κ*B/p65 nuclear translocation was also significantly increased in the glomeruli (Figures 7(a)–7(c)). After MRL/lpr mice were intraperitoneally injected with TAK242 for 8 weeks, MyD88 expression and NF-*κ*B/p65 nuclear translocation were downregulated (Figures 7(d) and 7(e)). Additionally, TAK242 also alleviated glycocalyx shedding (Figures 7(f)–7(h)) and downregulated the level of VCAM-1 and NO (Figures 7(i)–7(k)). In summary, extracellular HMGB1 mainly induced GEC injury by activating the TLR4/MyD88/NF-*κ*B/p65 pathway in LN.

## 4. Discussion

GECs are highly specialized cells characterized by transcellular fenestrations that allow small molecules to cross but limit protein diffusion. Glycocalyx covers both fenestral and interfenestral of the GEC surface and helps to regulate the vascular permeability and fluid balance and isolate blood cells from the vascular wall [17]. Under physiological conditions, the shedding and synthesis of glycocalyx keep a dynamic balance and the integrity of glycocalyx maintains the homeostasis of the normal vascular system, while the shedding of glycocalyx induces vascular permeability [18]. Syndecan-1 (SDC-1) is a core protein component of the glycocalyx on the GEC surface and an increased level of SDC-1 in the blood indicated glycocalyx shedding. As a transmembrane protein, its intracellular segment is connected to the cytoskeleton. The present research has shown that SDC-1 was elevated in many diseases such as sepsis, diabetes, trauma, and surgery [19, 20], and the degradation of glycocalyx was considered to be the cause of microcirculation dysfunction [21]. In our study, we found that SDC-1 was reduced in the renal glomeruli following by the thinner glycocalyx and elevated level in circulation. Importantly, the level of circulating SDC-1 was positively correlated with proteinuria, suggesting that glycocalyx shedding might be related to GFB dysfunction and proteinuria in LN.

Glycocalyx shedding uncovered membrane surface adhesion molecules such as VCAM-1, promoted the adhesion of leukocytes to the vessel wall [22], and induced the production of NO. VCAM-1 and NO have been reported to be significantly elevated in patients with LN and involved in the damage of endothelial cells [23–26]. In our experiments, the levels of VCAM-1 and NO were increased in the kidney of MRL/lpr mice and HRGECs treated with LN plasma, which was consistent with previous reports. In addition, peripheral blood VCAM-1 was positively correlated with SDC-1, indicating that glycocalyx shedding might facilitate the exposure of adhesion molecules and aggravate GEC injury. In this experiment, LN plasma treatment resulted in increased permeability of HRGEC monolayer and shedding of SDC-1, destroyed the intercellular junctions, and promoted the rearrangement of cytoskeletal, all of which indicated the injury of HRGEC and glycocalyx shedding in LN.

Extracellular HMGB1 is a proinflammatory mediator that participates in many inflammatory and autoimmune diseases [27]. Our previous studies have shown that the level of HMGB1 in serum was significantly increased in LN patients, and extracellular HMGB1 was not only located in mesangium but also located in endothelial cell of glomeruli. Other research has shown that HMGB1 could induce the cytoskeleton rearrangement of human pulmonary vascular endothelial cells and increase the permeability of endothelial cells [28, 29]. Therefore, to further investigate the role of HMGB1 in endothelial cell in LN, the GLY, an inhibitor of HMGB1, and anti-HMGB1 antibody were used. Our results showed that inhibition of HMGB1 significantly improved the hyperpermeability of HRGEC monolayer, alleviated the glycocalyx shedding, reduced the VCAM-1 and NO expression, and improved the intercellular tight junctions and cytoskeletal rearrangement induced by LN plasma. Meanwhile, the MRL/lpr mice treated with GLY showed similar results and endothelial cell injury of glomeruli was remarkably improved. In conclusion, extracellular HMGB1 was involved in the injury of GEC in LN.

In general, extracellular HMGB1 might activate downstream signaling pathways and lead to inflammation and cell injury by binding various receptors such as receptor for advanced glycation end products (RAGE) and Toll-like receptors (TLRs) [30, 31]. TLR4 is one of the crucial receptors of HMGB1; the persistent activation of TLR4 induces the damage of kidney, cardiovascular, and CNS tissue in hypertension [32]. In this study, the levels of TLR4, MyD88 expression, and the nuclear translocation of NF-*κ*B were significantly increased in GECs of MRL/lpr mice and HRGEC stimulated by LN plasma. Coincubation with TAK242 and/or GLY improved the injury of GEC and alleviated the glycocalyx shedding, downregulated the expression of MyD88, and reduced the nuclear translocation of NF-*κ*B, suggesting that extracellular HMGB1 induced the GEC injury by activation of TLR4. Our results also showed that inhibiting MyD88 and the nuclear translocation of NF-*κ*B partially mitigated the GEC injury and glycocalyx shedding. These results suggest that extracellular HMGB1 plays an important role in GEC injury in LN via activating the TLR4/MyD88/NF-*κ*B signaling pathway.

Unfortunately, there was no significant difference in proteinuria after MRL/lpr mice were treated with GLY or TAK242 compared to model mice. However, previous research showed that intravenous injection of anti-HMGB1 monoclonal antibody improved albuminuria in MRL/lpr mice [33], and TLR4 inhibitor reduced the level of serum creatinine in MRL/lpr mice [34]. These differences might be related to treatment duration, and/or podocytes might play a more important role than GECs in the occurrence of proteinuria, so solely alleviating GEC injury may not completely reverse GFB damage. This requires further research in the future.

## 5. Conclusion

In summary, the shedding of glomerular endothelial glycocalyx contributed to proteinuria in the pathogenesis of LN, and LN plasma stimulation induced GEC injury and increased the permeability of GECs. Functionally, extracellular HMGB1 induced the endothelial glycocalyx shedding and GEC injury in LN. The activation of TLR4 and its downstream signaling pathway in GECs is a possible underlying mechanism in HMGB1-induced dysfunction of GECs, which provide a potential therapeutic target for decreasing the progression of LN.

## Data Availability

No data were used to support this study.

## Abbreviations

LN: Lupus nephritis
GEC: Glomerular endothelial cell
SLE: Systemic lupus erythematosus
GFB: Glomerular filtration barrier
SDC-1: Syndecan-1
VCAM-1: Vascular cell adhesion molecule 1
HMGB1: High mobility group box 1
HRGECs: Primary human renal glomerular endothelial cells
IF: Immunofluorescence
IHC: Immunohistochemistry
DAPI: 4′,6-Diamidino-2-phenylindole
FITC-BSA: Fluorescein isothiocyanate-labeled bovine serum albumin
FBS: Fetal bovine serum
ELISA: Enzyme-linked immunosorbent assay
NO: Nitric oxide
TEM: Transmission electron microscopy
TLR: Toll-like receptor
TEER: Transendothelial electrical resistance
SDS-PAGE: Sodium dodecyl sulfate-polyacrylamide gel electrophoresis
PVDF: Polyvinylidene difluoride
ANOVA: One-way analysis of variance
GLY: Glycyrrhizin
RAGE: Receptor for advanced glycation end products.
